# The interplay between electrical and chemical synaptogenesis

**DOI:** 10.1152/jn.00398.2018

**Published:** 2018-08-01

**Authors:** Shaista Jabeen, Vatsala Thirumalai

**Affiliations:** ^1^National Centre for Biological Sciences, Tata Institute for Fundamental Research, Bangalore, India; ^2^Manipal Academy of Higher Education, Madhav Nagar, Manipal, India

**Keywords:** activity, circuit assembly, connexin, development, dye coupling, electrotonic coupling, gap junctions, innexin

## Abstract

Neurons communicate with each other via electrical or chemical synaptic connections. The pattern and strength of connections between neurons are critical for generating appropriate output. What mechanisms govern the formation of electrical and/or chemical synapses between two neurons? Recent studies indicate that common molecular players could regulate the formation of both of these classes of synapses. In addition, electrical and chemical synapses can mutually coregulate each other’s formation. Electrical activity, generated spontaneously by the nervous system or initiated from sensory experience, plays an important role in this process, leading to the selection of appropriate connections and the elimination of inappropriate ones. In this review, we discuss recent studies that shed light on the formation and developmental interactions of chemical and electrical synapses.

## INTRODUCTION

Information processing within nervous systems depends on connectivity between neurons. It is now well appreciated that connectivity in the nervous system is mediated by two classes of synapses: electrical and chemical. Electrical synapses are neuronal gap junctions forming intercellular channels through which ions and small metabolites can pass. These channels are formed by the apposition of connexin (Cx) proteins in vertebrates and innexin (inx) proteins in invertebrates. Chemical synapses, on the other hand, are formed by the apposition of a presynaptic terminal and a postsynaptic membrane separated by a synaptic cleft. Neurotransmitters released from the presynaptic terminal bind to cognate receptors on the postsynaptic side, leading to electrical and/or chemical signaling on the postsynaptic side.

Nearly a hundred years ago, the nature of connectivity in the nervous system was the subject of an intense “soup vs. spark” debate referring to chemical and electrical transmission, respectively (Cowan and Kandel 2001). Toward the beginning of the twentieth century, multiple lines of evidence from the peripheral nervous system pointed to the existence of chemical neurotransmission. Notable among these was the classical experiment of Otto Loewi in 1921 demonstrating that vagus nerve stimulation inhibited heartbeat by releasing “vagusstoff” (Loewi 1924), later identified to be acetylcholine (Cowan and Kandel 2001). Subsequently, the discovery of the ionic basis and quantal nature of neurotransmission at the neuromuscular junction (Fatt and Katz 1951, 1952) and John Eccles’s demonstration of synaptic inhibition in the spinal cord (Brock et al. 1952) provided stronger proof for the existence of chemical neurotransmission across synaptic junctions in the central nervous system (CNS) and in the periphery. These discoveries led to the view that all transmission was likely to be chemical, and the debate was almost settled in favor of the soup. However, Furshpan and Potter, recording from the crayfish giant axonal fiber-to-motor neuron synapse (Furshpan and Potter 1957, 1959), and Akira Watanabe, recording from the lobster cardiac ganglion (Watanabe 1958), demonstrated the existence of electrical transmission. Electrical transmission was then discovered in goldfish (Furshpan 1964), puffer fish (Bennett et al. 1967), and, later, in the mammalian brain (Baker and Llinás 1971). As a result of work over the last forty years, we now understand that both chemical and electrical transmission are widely prevalent in nervous systems and that interactions between the two shape neuronal response (Connors 2017; Nagy et al. in press; Pereda 2014; Szczupak 2016). Furthermore, these two modes of transmission play critical roles during the development and assembly of neural circuits (Baker and Macagno 2016; Pereda 2014). In this review, we discuss our current understanding of the formation of electrical and chemical synapses and how these two types of synapses regulate each other during development.

## THE FORMATION OF CHEMICAL SYNAPSES

Chemical synaptogenesis has received extensive attention, and we now understand several of the key steps in the formation of synapses (for a comprehensive review, see Jin 2002; Waites et al. 2005). Much of the work has focused on glutamatergic excitatory synapses, although some light has also been thrown on inhibitory synaptogenesis (see Dobie and Craig 2011; Kuzirian et al. 2013; Wierenga 2017; Woo et al. 2013).

### Target Selection

When an axon reaches a target region there are several postsynaptic partners to choose from, yet synapses are formed only with specific cells. For example, neurons do not make synapses with glia or connective tissue or with themselves (autapses) in vivo. The early selection of appropriate targets with which to make synapses is in part mediated by signaling mechanisms that also mediate axon guidance such as the calcium-dependent cell adhesion molecules cadherins and protocadherins (de Wit and Ghosh 2016; Waites et al. 2005). Several studies have shown their presence at initial axo-dendritic contact sites, and they mediate cell adhesion via mainly homophilic interactions (Fannon and Colman 1996; Hayashi and Takeichi 2015; Takeichi et al. 1997; Uchida et al. 1996; Weiner et al. 2005). Because of their specific expression patterns across the CNS and the diversity of their isoforms, these classes of molecules are ideally suited for recognition of the correct target sites, at least at the cell type level. Thus these early signaling events help in target recognition and stabilization of the initial contact site.

### Synapse Formation

Once a membrane contact is formed, other cell adhesion molecules like synaptic cell adhesion molecules (SynCAMs) and neuroligins can initiate presynaptic specialization. Neuroligins are present on the postsynaptic membrane and bind to presynaptic neurexins. Whereas neuroligins induce the clustering of vesicles and assembly of other presynaptic components, neurexins, on the other hand, induce the clustering of NMDA receptors (NMDARs) and PSD-95 at excitatory synapses and GABA_A_ receptors and Gephyrin at inhibitory synapses (Dean et al. 2003; Dean and Dresbach 2006; Graf et al. 2004; Scheiffele et al. 2000). SynCAMs, members of the immunoglobulin family adhesion molecules, are expressed on both the pre- and postsynaptic sides and signal via homophilic or heterophilic interactions (Biederer et al. 2002; Fogel et al. 2007). SynCAM1 has been shown to accumulate at axo-dendritic contact sites within 5 min after first contact (Stagi et al. 2010) and induce the assembly of presynaptic active zone machinery (Biederer et al. 2002). Besides these membrane-bound adhesion molecules, secreted proteins like Wnts, fibroblast growth factors, Narp, and EphrinB have also been demonstrated to induce postsynaptic receptor clustering and/or formation of the presynaptic active zone (Dalva et al. 2000; Lee et al. 2017; Mi et al. 2002; O’Brien et al. 1999; Scheiffele 2003).

### Synapse Maturation

Nascent synapses that have formed after these signaling events undergo a process of maturation during which synaptic vesicles and postsynaptic receptors increase in number in a coordinated fashion and dendritic spines are formed. During this time, several presynaptic and postsynaptic changes occur, leading to the sculpting of synaptic responses. Changes in the probability of transmitter release and quantal size have been reported (Cline and Haas 2008; Garner et al. 2006; Vaughn 1989; Waites et al. 2005; Ziv and Garner 2004). Likewise, “silent” synapses—synapses conducting solely via NMDARs—could be unsilenced by the insertion of AMPA receptors (Petralia et al. 1999; Wu et al. 1996). NMDARs themselves could undergo changes in receptor subunit composition, resulting in alterations in the postsynaptic response (Law et al. 2003; Tovar and Westbrook 1999).

Neuronal activity, induced by neurotransmitter release, triggers a wide range of maturational processes. Indeed, the excitatory neurotransmitter glutamate and the inhibitory neurotransmitter GABA have been shown to promote dendritic spinogenesis and AMPA receptor clustering (Kwon and Sabatini 2011; Oh et al. 2016). In addition, GABA also induces the clustering of the inhibitory synaptic scaffold molecule gephyrin, leading to the formation of functional inhibitory synapses (Oh et al. 2016). From these studies, we now know that the elevation of intracellular calcium caused by these two neurotransmitters is critical for activity-dependent synaptogenesis. In the case of glutamate calcium elevation is mediated by NMDARs, whereas in the case of GABA its depolarizing action in early development causes calcium entry via voltage-dependent channels.

Activity-dependent signaling is also critical for synapse elimination, the process by which circuits are fine-tuned during development. This process has been shown to occur in the CNS and in the periphery (Purves and Lichtman 1980). Muscle fibers are innervated by multiple motor neurons at birth; however, activity-dependent synaptic competition ensures the survival of only one innervation, with the elimination of all others. Weak synapses are further weakened and eliminated while strong ones are retained (Balice-Gordon and Lichtman 1994; Buffelli et al. 2003). A similar process occurs in the cerebellum, where multiple climbing fibers innervate Purkinje neurons at birth and subsequently all but the strongest input are eliminated, such that in adults each Purkinje neuron is innervated only by a single climbing fiber (Hashimoto and Kano 2013). A number of players have been shown to regulate this process, including calcium entry via voltage-dependent calcium channels (Hashimoto et al. 2011), brain-derived neurotrophic factor-TrkB signaling (Bosman et al. 2006; Johnson et al. 2007), and the activation of parallel fiber pathways (Hashimoto and Kano 2013). Electrical activity that recruits the above-mentioned signaling pathways could likely be the central regulator of this process.

Several brain regions manifest spontaneous neural activity in the form of periodic depolarizations that propagate through the network and aid in electrical activity-dependent synaptogenesis (Blankenship and Feller 2010; Kirkby et al. 2013; O’Donovan et al. 1998). The mechanisms of origin and propagation of spontaneous activity in nascent networks and its influence on synaptogenesis is a vast area, and several excellent recent reviews have addressed this topic (Blankenship and Feller 2010; Hanson et al. 2008; Kirkby et al. 2013; O’Donovan et al. 1998; Spitzer 2012; Vonhoff and Keshishian 2017). In addition, sensory experience plays a critical part in sculpting circuits by the induction and stabilization of new synapses (Aizenman and Cline 2007; Haas et al. 2006; Ruthazer et al. 2006; Sin et al. 2002), a process that also seems to be recruited in mature animals (Knott et al. 2002; Trachtenberg et al. 2002; Zito and Svoboda 2002).

## THE FORMATION OF ELECTRICAL SYNAPSES

In comparison to the wealth of information available regarding chemical synaptogenesis, precious little is known about the formation of electrical synapses. Electrical synapses require the coordinated trafficking of thousands of connexin or innexin proteins to the junctional area of the two coupled neurons. How do the coupled neurons direct the trafficking of gap junctional proteins to the junction where the two membranes come in contact? What signaling mechanisms may be involved in this process? Some knowledge about the processes involved has been gleaned from in vitro assays involving connexins found within and outside the nervous system.

### Clues from Nonneural Connexin Gap Junctions

In vertebrates, connexin subunits first oligomerize into connexons or hemichannels. The docking of connexons from two neurons leads to the formation of a functional gap junction. Thousands of such gap junctional channels form a plaque at electrical synapses (Leitch 1992). What are the mechanisms by which electrical synapses are assembled? Clues regarding the molecular signaling events leading to electrical synapse formation can be obtained from observations on gap junction plaque assembly or turnover in nonneural tissues.

Cx43, one of the most commonly occurring connexins, is important for diverse functions such as the propagation of the cardiac action potential for synchronizing heartbeat, the coordinated contractions of uterine smooth muscles during birth, and the coupling of astrocytes in the brain (Goodenough and Paul 2009; Tong et al. 2009). The synthesis and trafficking of Cx43 have been well studied in these cell types (Epifantseva and Shaw 2018). From these studies in vitro and in vivo, it is now known that this process involves the rapid transcription and accumulation of Cx43 mRNA in the cytoplasm, translation of the mRNA to protein in the rough endoplasmic reticulum, trafficking to the Golgi and assembly into connexons, vesicular transport along actin microfilaments and microtubules, insertion into the plasma membrane, and, finally, docking to cognate hemichannels at gap junctional plaques.

The above steps are regulated by multiple signaling events. For instance, transcription of Cx43 is tightly regulated in myometrial cells by the balance between estrogen and progesterone signaling. Cx43 transcripts are virtually undetectable in the myometrium of nonpregnant rats; however, they gradually increase after day 10 of pregnancy. Cx43 transcript and protein levels are maximal around the time of delivery, decreasing to baseline levels in the days following parturition (Hendrix et al. 1995; Lye et al. 1993). This timeline of Cx43 synthesis mirrors the formation of functional gap junctional coupling among the myometrial cells around the time of parturition (Garfield et al. 1977).

### Transcriptional Regulation of Neural Connexins

The above results from nonneural connexins suggest that connexin protein synthesis could be a point of regulation for the assembly of electrical synapses in the nervous system. Indeed, in rodents the expression of Cx36, known to form electrical synapses, increases during the first two postnatal weeks—stages at which gap junctional coupling among cortical neurons increases. The expression falls off during the third and fourth weeks, at which stages gap junctional coupling among cortical neurons also decreases (Belluardo et al. 2000; Belousov and Fontes 2013; Condorelli et al. 2000; Montoro and Yuste 2004). Similarly, in teleosts the orthologous Cx35 gene expression is activated before dye coupling is observed among hindbrain neurons (Jabeen and Thirumalai 2013). What could be the trigger for the activation of transcription of Cx35/36? Again, clues are offered in nonneural tissues. Cx36 also mediates gap junctional coupling between insulin-producing β cells of the pancreas. In these cells, Cx36 expression is activated by the binding of NeuroD1 (also called β2) to E-box elements in the promoter region of the gene (Nlend et al. 2012). NeuroD1 is widely expressed within the CNS and is involved in the functional differentiation of neurons (Cho and Tsai 2004; Kawakami et al. 1996). It is tempting to speculate that NeuroD1/β2 could trigger a common transcriptional program to activate gap junction formation in two different tissues. It is also likely that in different neuronal subtypes distinct transcription factors drive the expression of Cx35/36 at appropriate developmental stages.

### Role of Intracellular Trafficking

The trafficking and insertion of neural connexons into gap junctional plaques are also steps that could be regulated during electrical synapse formation. First, connexins need to oligomerize to form connexons, and there is some variation depending on the connexin isotype, whether this process occurs in the endoplasmic reticulum or Golgi (Diez et al. 1999; Evans et al. 1999; Musil and Goodenough 1993; Solan and Lampe 2005). The trafficking of connexons on vesicles from the Golgi to the plasma membrane is also connexin dependent, with some forms using actin microfilaments and some getting trafficked on both actin microfilaments and microtubules (Thomas et al. 2001).

Stable intercellular conducting channels can be formed in *Xenopus* oocyte pairs when connexin RNA is injected into them and when the two cells are brought in contact. This has been shown for both neural and nonneural connexins (Barrio et al. 1991; Dahl et al. 1987; Ebihara et al. 1989; Swenson et al. 1989). Does the cell-cell contact initiate signaling pathways leading to the trafficking of the expressed connexins to the junctional site? If so, what might these signaling pathways be? We still do not have a clear understanding of these pathways, yet a recent study implicated Neurobeachin (Nbea), a protein known to be involved in intracellular trafficking, in the formation of electrical synapses (Miller et al. 2015). Nbea is expressed at high levels in the brain, and mutations in this protein are associated with the occurrence of autism (Castermans et al. 2003). Nbea has been shown to be important for neuromuscular and CNS synaptogenesis in vertebrates and invertebrates (Farzana et al. 2016; Medrihan et al. 2009; Nair et al. 2013; Niesmann et al. 2011; Su et al. 2004; Volders et al. 2012). The study by Miller et al. focused on a circuit mediating fast escapes in larval zebrafish, i.e., the Mauthner circuit. Mauthner neurons are located bilaterally in the hindbrain and send a contralateral axonal projection to the spinal cord, where they make electrical synapses with commissural local (CoLo) interneurons in every spinal segment. CoLo interneurons are glycinergic inhibitory and act to lateralize the escape response (Satou et al. 2009). Importantly, CoLo pairs in every segment make inhibitory synapses onto each other. Miller and colleagues showed that these glycinergic synapses were disrupted in Nbea mutant zebrafish. The electrical synapse between Mauthner neurons and CoLos is asymmetric, with Cx35.5 on the Mauthner side and Cx34.1 on the CoLo side (Miller et al. 2017). Mauthner-CoLo neuron electrical synapses were defective in Nbea mutants, showing weak dye coupling and decreased connexin localization. Staining for the electrical synapse scaffold protein zonula occludens-1 (ZO-1) and the glycinergic and GABAergic synapse scaffold protein Gephyrin was also reduced in these mutant fish. Taken together, these results demonstrate that Nbea is required for electrical and chemical synaptogenesis. In a series of elegant experiments, Miller and colleagues tested the requirement of Nbea in Mauthner vs. CoLo neurons for the formation of electrical synapses by transplanting cells between wild-type and mutant fish, thus creating mosaic animals. Expression of Nbea only in the CoLo neuron was sufficient to rescue Mauthner-CoLo electrical synapse, suggesting that Nbea functions in the trafficking of Cx34.1. Likewise, in CoLo pairs Nbea was required in the postsynaptic CoLo for the trafficking of glycinergic receptors to synaptic sites. Restoring Nbea in the postsynaptic CoLo alone was sufficient to rescue glycinergic synaptogenesis. These results together suggest that Nbea may play a common but specific role in the trafficking of membrane and scaffold proteins at electrical and chemical synapses.

### Scaffolding Molecules and Electrical Synaptogenesis

What mechanisms might control the accretion of gap junctional hemichannels to specific spots on the membrane to form junctional plaques? Although early events during de novo gap junction assembly have so far not been described, several molecular players have been implicated in gap junction channel turnover at preexisting plaques. Both Cx43 and Cx35/36 are inserted into the plasma membrane in nonjunctional areas, usually at the periphery of preexisting junctions (Flores et al. 2012; Gaietta et al. 2002; Lauf et al. 2002). These then move laterally within the membrane into junctional plaques, where they dock on hemichannels from the partner cell to form functional channels. Scaffolding molecules that tether connexins to the cytoskeleton are likely to be involved in stabilizing newly inserted connexins in junctional plaques. Organization of the plaque could be driven by scaffolding molecules that bind connexins at their COOH termini and dock them to cytoskeletal elements lying underneath the junctional areas. Several scaffold molecules are known to associate with gap junctional plaques such as tight junction protein-1 (tjp1) or ZO-1 (Flores et al. 2008; Li et al. 2004; Lynn et al. 2012). Tjp1 is a member of the MAGUK family and interacts with connexins at their PDZ domains (Giepmans and Moolenaar 1998; Li et al. 2004). In zebrafish, where there are two copies of tjp1, a and b, tjp1b was shown to be essential for the formation of Mauthner-CoLo electrical synapses. Although expressed widely within the zebrafish CNS, Tjp1b was found very close to the Cx34.1 channels at the plaque. Similarly to Nbea, Tjp1 is also required on the postsynaptic side, i.e., in the CoLo neuron for the assembly of this junction. In chimeric animals, when Tjp1b was absent in the CoLo neurons Cx35.5/Cx34.1 junctions failed to form. However, when it was absent in Mauthner neurons the electrical junctions were formed, indicating that the requirement for Tjp1b is asymmetric and specific. This result also suggests that the postsynaptic Tjp1b can organize the presynaptic Cx35.5 via its interactions with the Cx34.1. On the other hand, Cx35.5 hemichannels are unable to exert such an organizing influence on the Cx34.1 hemichannels in the absence of Tjp1b (Marsh et al. 2017). These results point to the highly specific nature of regulation of connexin hemichannels by scaffolding molecules.

Apart from Tjp1, other scaffold molecules that are known members of tight and adherens junctions, such as AF-6, MUPP1, and Cingulin, have also been found at neuronal and nonneuronal gap junctional plaques (Lynn et al. 2012). This suggests a common molecular plan for the construction of intercellular junctions, be it tight junctions, adherens junctions, or gap junctions, in a variety of tissues. If this were the case, vital clues regarding the assembly and organization of electrical synapses can be gleaned by taking a holistic approach to the study of intercellular junctions.

## INTERPLAY BETWEEN ELECTRICAL AND CHEMICAL SYNAPSES

It is well established in several systems that gap junctional coupling increases in the nervous system in the period immediately preceding stages at which chemical synaptogenesis occurs (Connors et al. 1983; Fischbach 1972; Jabeen and Thirumalai 2013; Lopresti et al. 1974). In the developing neocortex of rat, expression of Cx36 has been shown to increase from postnatal day (P)0 to P14, and the incidence of dye coupling among neurons also increases during this period, suggesting an increase in electrotonic coupling among neurons. Thereafter, it continues to decline until a steady state is reached in the adult stages (Belluardo et al. 2000). Such a pattern of transient increase followed by decrease in Cx36 expression and gap junctional coupling in the neocortex and other brain regions during late embryonic/postnatal development is seen in mice (Söhl et al. 1998) and ferrets (Montoro and Yuste 2004) also. The decrease in electrical coupling occurs concomitantly with an increase in chemical synaptic transmission, suggestive of interplay between the two. Indeed, reports of interactions between electrical and chemical synaptogenesis are emerging.

### Role of Electrical Synapses in Chemical Synaptogenesis

Multiple studies implicate electrical synapses in the development of chemical synapses in vertebrate (Maher et al. 2009; Yu et al. 2012) and invertebrate (Curtin et al. 2002; Szabo and Zoran 2007) nervous systems. When Cx36 was knocked out in mice, excitatory synapses between olfactory bulb mitral cells failed to form (Maher et al. 2009). On the other hand, in the thalamus the number of inhibitory synapses onto thalamic relay neurons increased, with a concomitant decrease in their dendritic complexity (Zolnik and Connors 2016). Perhaps the strongest evidence that gap junctions are vital for chemical synaptogenesis was obtained in leeches by knocking down the invertebrate gap junction protein inx1. In adult leeches, mechanosensitive P cells are connected to AP cells via strong excitatory chemical synapses and weak electrical coupling. In embryonic leeches, this connection is purely electrical. Could it be that the electrical synapse in embryonic leeches guides the formation of the chemical synapse between the P and AP cells? Injecting double-stranded RNA (dsRNA) targeting inx1 transcripts during early embryonic development led to decreased P-to-AP excitatory synaptic strength in juveniles, long after the knockdown effect of the inx1-dsRNA had worn off (Todd et al. 2010). Another example of this phenomenon is seen in the formation of specific synaptic connections between pyramidal neurons in the neocortex of mice. A recent elegant study showed that neurons that are derived from the same radial glial cell (“sisters”) are preferentially electrically coupled (Yu et al. 2012) and go on to form strong excitatory connections with each other (Yu et al. 2009). When these Cx26-mediated electrical synapses were disrupted, the chemical synaptic connections failed to form (Yu et al. 2012). Taken together, these results suggest that electrical synapses during early development are critical for the specification of chemical synapses. Gap junctions may aid chemical synaptogenesis by bringing prospective synaptic partners in close contact, by increasing correlations in activity, and by transfer of signaling molecules from one cell to the other.

### Regulation of Electrical Synapses by Chemical Synapses

Chemical neurotransmission can also regulate gap junctional communication in developing networks through distinct pathways. The neurotransmitters glutamate and GABA can cause increase and decrease, respectively, in gap junctional coupling at different developmental stages by triggering distinct signaling cascades. In the rodent hypothalamus and cortex, Cx36-mediated gap junctions are upregulated starting from P0 until P15 via the activation of group II metabotropic receptors (mGluRs). Conversely, activation of GABA_A_ receptors prevents such developmental upregulation of Cx36 expression and gap junctional coupling. Binding of glutamate to group II mGluRs activates cAMP/PKA-dependent signaling cascades and increases Cx36 expression (Park et al. 2011). Cx36 expression is under the control of a neuron-restrictive silencing element (NRSE), which normally suppresses transcription (Martin et al. 2003). Activation of group II mGluRs was shown to lift the NRSE-mediated suppression of Cx36 transcription (Park et al. 2011).

In contrast, the developmental uncoupling of gap junctions is regulated by a distinct set of signaling cascades but triggered by the same neurotransmitter, glutamate. From P7 to P21 gap junctions are progressively uncoupled, and this period overlaps with major chemical synaptogenesis in the hypothalamus as well as in the spinal cord, hippocampus, neocortex, and striatum. Such uncoupling is mediated by the activation of NMDARs (Arumugam et al. 2005; Mentis et al. 2002), resulting in signaling via CaMKII/IV and PKC-dependent pathways, leading to activation of the phosphoprotein cAMP response element binding protein (CREB). CREB is known to regulate the expression of multiple genes including Cx36 by binding to a Ca^2+^/cAMP response element within the promoter region and likely causes developmental uncoupling directly by reducing the number of Cx36 transcripts (Arumugam et al. 2005). Thus distinct signaling cascades and transcriptional regulatory pathways triggered by chemical neurotransmission control the developmental increase and decrease of Cx36 gap junctions.

### Role of Sensory Experience

Sensory experience during early life stages can be a critical factor in the developmental decrease of gap junctional coupling. In newborn mice, gap junctional coupling between mitral cells is high at birth and is decreased significantly by 1 mo after birth, coincident with the emergence of chemical synaptic coupling between these cells. Unilateral sensory deprivation through suturing of the nostril inhibited the developmental decrease in junctional coupling on the deprived side alone (Maher et al. 2009). This result shows that sensory experience during development is a key regulator of electrical synapses among neurons. This likely occurs through sensory input-mediated activation of NMDARs and the subsequent loss of junctional coupling as described above.

As a parallel to the chemical synapse-mediated loss of electrical coupling, electrical synapses are key regulators of developmental chemical synapse elimination. It has been suggested that transient electrical synapses could act to increase correlated firing among neurons, thus aiding activity-dependent chemical synaptogenic processes. Correlated activity could also play a role in synapse elimination, a critical step in the assembly of neural circuits (see *Synapse Maturation*). Indeed, the presence of Cx40-mediated gap junctions increases correlated firing of motor neurons in the neonatal mouse spinal cord. Cx40 expression and electrotonic coupling among motor neurons progressively decrease over the first two postnatal weeks (Personius et al. 2007). During this time, temporal correlations in the firing of motor neurons also decrease (Personius and Balice-Gordon 2001). In mice lacking Cx40, motor neurons show poor correlation in firing even at birth, and synapse elimination is accelerated (Personius et al. 2007). This shows that gap junctions are critical for maintaining correlated firing among motor neurons in the neonatal cord and therefore act to oppose competition among these neurons at the neuromuscular junction. The developmental downregulation of gap junctions serves to decrease correlated firing, resulting in synaptic competition and subsequent elimination of ineffective inputs at the neuromuscular junction. Interestingly, the uncoupling of motor neurons is mediated by NMDAR-mediated neurotransmission—blockade of NMDARs during the first postnatal week results in maintenance of electrical coupling between motor neurons (Mentis et al. 2002). These findings underline the fact that electrical and chemical synaptogenesis are tightly interlinked and that during development the two processes constantly regulate each other to ensure proper circuit assembly.

## CONCLUSIONS

Electrical and chemical synapses function not in isolation but as constantly interacting entities in the nervous system (Pereda 2014). Therefore it is not surprising that the formation of these two classes of synapses is regulated by interlinked processes. We are just beginning to understand the molecular mechanisms that mediate the interactions between developing electrical and chemical synapses. With the emergence of genome editing tools (Doudna and Charpentier 2014), it has now become possible to genetically delete particular molecular players and ask how electrical and chemical synaptogenesis are affected. Furthermore, the ability to image and manipulate activity in entire brains of small model organisms (Ahrens and Engert 2015) also presents the opportunity to follow the effects of these alterations at the circuit, systems, or behavioral levels. Armed with this toolkit, neuroscientists can, now more than ever, begin to decipher mechanisms that coregulate electrical and chemical synaptogenesis.

## GRANTS

The authors acknowledge the following sources of funding support: the Wellcome Trust-DBT India Alliance Intermediate Fellowship (V. Thirumalai); Department of Biotechnology (V. Thirumalai), Science and Engineering Research Board, Department of Science and Technology (V. Thirumalai), and Department of Atomic Energy (V. Thirumalai), Government of India; and the Council for Scientific and Industrial Research-UGC Fellowship (S. Jabeen).

## DISCLOSURES

No conflicts of interest, financial or otherwise, are declared by the authors.

## AUTHOR CONTRIBUTIONS

S.J. and V.T. drafted manuscript; S.J. and V.T. edited and revised manuscript; S.J. and V.T. approved final version of manuscript.
